# Ursodeoxycholic acid as adjuvant treatment to phototherapy for neonatal hyperbilirubinemia: a systematic review and meta-analysis

**DOI:** 10.1007/s12519-022-00563-z

**Published:** 2022-06-11

**Authors:** Ilari Kuitunen, Panu Kiviranta, Ulla Sankilampi, Marjo Renko

**Affiliations:** 1 Institute of Clinical Medicine, Department of Pediatrics, University of Eastern Finland, Kuopio, Finland; 2 Department of Pediatrics, Mikkeli Central Hospital, Porrassalmenkatu 35-37, 50100 Mikkeli, Finland; 3 Department of Pediatrics, Kuopio University Hospital, Kuopio, Finland

**Keywords:** Jaundice, Phototherapy, Unconjugated hyperbilirubinemia, Ursodeoxycholic acid

## Abstract

**Background:**

Neonatal hyperbilirubinemia is observed in most newborns, and 5–15% of neonates require phototherapy. Phototherapy is effective but often prolongs hospitalization and has both short-term and potential long-term harms. The aim of this systematic review and meta-analysis was to evaluate the role of ursodeoxycholic acid (UDCA) combined with phototherapy in neonatal hyperbilirubinemia.

**Methods:**

A literature search was conducted on September 1, 2021; 590 studies were screened, and 17 full texts were assessed by two authors. We included randomized controlled trials with or without placebo intervention. Primary outcomes were changes in total bilirubin levels at 24 hours and phototherapy duration. We calculated mean differences with 95% confidence intervals (CI).

**Results:**

Six studies with 880 neonates were included. Of these studies, only two used a placebo-controlled double-blinded design. The overall risk of bias was high in one and moderate in four of the included studies. The mean decrease in the total bilirubin level during the first 24 hours was 2.06 mg/dL (95% CI 0.82–3.30; six studies) greater in the UDCA treatment group. The phototherapy duration was 19.7 hours (95% CI 10.4–29.1; five studies) shorter in the UDCA treatment group.

**Conclusions:**

We found low-quality evidence that UDCA as an adjuvant to phototherapy seems to decrease total bilirubin faster and shorten phototherapy duration compared to standard treatment. Further studies are needed to confirm the efficacy, acute and long-term outcomes, and safety before implementing UDCA as an adjuvant to phototherapy in neonatal hyperbilirubinemia.

**Supplementary Information:**

The online version contains supplementary material available at 10.1007/s12519-022-00563-z.

## Introduction

Neonatal hyperbilirubinemia is a common finding, as approximately 50% of term neonates and 80% of preterm neonates develop hyperbilirubinemia. Approximately 10% of infants show increased levels of bilirubin up to 1 month of age [1, 2]. Between 5% and 15% of neonates require close monitoring and phototherapy, which is typically initiated at 2–5 days postnatally [1, 3, 4]. The indication for phototherapy is a rapidly rising or high serum total bilirubin level [5, 6], and the aim is to prevent neurotoxicity caused by unconjugated free bilirubin that crosses the blood-brain barrier.

Phototherapy was introduced 60 years ago [7], and it has remained the standard treatment for neonatal hyperbilirubinemia [8]. If bilirubin levels continue to rise despite phototherapy, exchange transfusion might be needed to treat severe hyperbilirubinemia. The typical duration of phototherapy is between 12 and 48 hours [9]. Phototherapy is used widely, and in addition to prolonged hospitalization, short-term harms include erythematous rash, retinal damage, irritability, loose stools, dehydration, feeding difficulties and the “bronze-baby” syndrome [10, 11]. Recently, the potential long-term harms of neonatal phototherapy have been discussed, as phototherapy has been associated with slightly increased rates of infant and childhood cancer [12, 13], the number of melanocyte nevi [14] and epileptic convulsions during childhood [15, 16].

Potential pharmacological therapies for unconjugated hyperbilirubinemia have gained interest, both to reduce lengths of hospital stays and to avoid more intensive therapies and their harmful side effects, such as those seen with exchange transfusions. A few studies have evaluated whether ursodeoxycholic acid (UDCA) would be effective as an adjuvant therapy [17–24]. UDCA is a bile acid, and it has been hypothesized to work by preventing the reabsorption of bilirubin from the intestines and thus occupying enterohepatic circulation [25, 26]. Although UDCA is an off-label treatment in neonates, it is widely used in conjugated hyperbilirubinemia and liver disorders [27–29]. UDCA is generally well tolerated [27]. UDCA was reported to be effective in reducing the duration of phototherapy in healthy term neonates [17–21], in sick neonates [23] and among neonates with G6PD deficiency [24]. One previous study found no additional value of combining UDCA with standard phototherapy [22].

The aim of this systematic review and meta-analysis is to analyze the effect of UDCA as an adjuvant to phototherapy in neonates with unconjugated hyperbilirubinemia.

## Methods

### Search strategies

The databases searched in this systematic review were PubMed (MEDLINE), the Cochrane Central Register of Controlled Trials (CENTRAL), Web of Science and Scopus. The literature search was conducted on September 1, 2021. The following phrase was used in the search: (“ursodeoxycholic acid”) AND (neonat* OR newborn*) AND (jaundice* OR bilirubin* OR phototherap*). We used neither language nor time restrictions. The results were then uploaded to the Covidence software (Covidence, Melbourne, Australia).

### Inclusion and exclusion criteria

All randomized controlled trials with or without placebo and regardless of blinding were included. Reports had to focus on UDCA use on newborns, but those including sick neonates, conjugated hyperbilirubinemia or only glucose-6-phosphate dehydrogenase deficiency (G6PD) were excluded. If newborns with more intensive hemolysis, such as Rh immunization or ABO incompatibility, were included in trials, randomization needed to be stratified to prevent imbalance between treatment groups. We had no exclusion criteria regarding prematurity or birthweight in our review.

### Review process

**Fig. 1:**
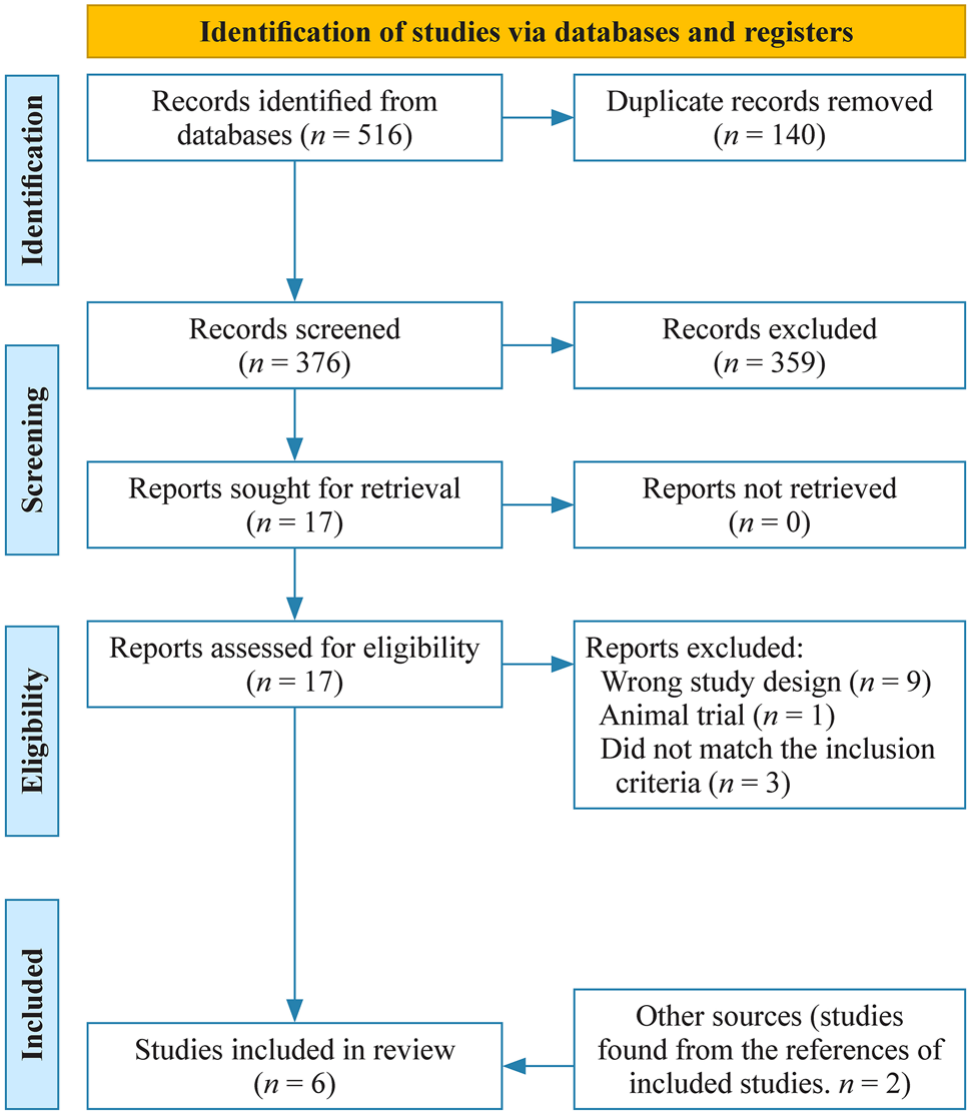
PRISMA flowchart of the review process. *PRISMA* preferred reporting items for systematic reviews and meta-analyses

Two authors (KI and KP) individually screened the abstracts, and conflicts were resolved by a third author (RM) or mutual consensus. Full texts were then assessed by two authors (KI, KP), and data were extracted using the Covidence 2.0 data extraction templates. The risk of bias was assessed according to the Cochrane tool for assessment by one author (KI), and a senior author (RM) was consulted if needed [30]. The risk of bias is reported in the Cochrane Risk of Bias 2.0 table, and it is presented by generating plots with the Robvis package [31]. Reporting quality was assessed using the Grading of Recommendations Assessment, Development and Evaluation methodology [32]. Background information on studies and study populations are presented in tables. A flowchart of the study process is presented in Fig. 1.

### Outcome measures

Our primary outcomes were changes in the total bilirubin level 24 hours after the initiation of phototherapy and phototherapy treatment duration. Secondary outcomes were return to hospital after discharge, harms of the treatment and cost-effectiveness. A subgroup analysis of preterm neonates was planned to be conducted if information was available.

### Statistics

Review Manager version 5.4 (the Cochrane Collaboration, London, UK) was used for the meta-analysis. Data analyses were performed according to the Cochrane handbook of systematic review guidelines. We calculated mean differences for continuous outcomes, as all the included studies used the same continuous outcome measurements. Risk ratios would have been calculated for dichotomous outcomes. Forest plots are presented for primary outcomes. The inconsistency index statistic *I*^2^ for heterogeneity was conducted, and if *I*^2^ > 50%, a random effect model was used. If heterogeneity was low (< 50%), the fixed effect model was chosen.

All the included studies reported a baseline level of bilirubin and post-intervention level in mg/dL and had standard deviations (SD) reported. However, only two studies reported the absolute mean change with SD. Therefore, we had to calculate the SD for change, as described in the Cochrane handbook, chapter 6.5.2.8 [33]. We decided to use the method in which one of the included studies is used for the calculation of the correlation coefficient. The correlation coefficient describes how similar the baseline and post-intervention measurements were across participants. We selected the work of Shahramian et al. [19], as in that work, the correlation coefficients were above 0.5 in both the treatment and control groups. If the correlation coefficient is below 0.5, post-intervention measures can be presented and interpreted directly. As the coefficient was above 0.5, we used the measured change from the baseline in reporting. The following formula was used for the calculation of the correlation coefficient.

$${\text{Corr}}_{{\text{E}}} = \frac{{{\text{SD}}_{{\text{E,baseline}}}^{2} + {\text{SD}}_{{\text{E,baseline}}}^{2} - {\text{SD}}_{{\text{E,change}}}^{2} }}{{2 \times {\text{SD}}_{{\text{E,baseline}}} \times {\text{SD}}_{{\text{E,final}}} }}$$

The mean of correlation coefficients, 0.73 (treatment group 0.83 and control group 0.63), calculated from Shahramian et al. [19], was used in the following formula to calculate the SD for mean change from baseline 24 hours after the initiation of phototherapy.

$${\text{SD}}_{{\text{E,change}}} = \sqrt {{\text{SD}}_{{\text{E,baseline}}}^{2} + {\text{SD}}_{{\text{E,final}}}^{2} - (2 \times {\text{Corr}} \times {\text{SD}}_{{\text{E,baseline}}} \times {\text{SD}}_{{\text{E,final}}} )}$$

### Protocol registration

This systematic review and meta-analysis has been reported according to the Preferred Reporting Items for Systematic Reviews and Meta-Analyses [34] (Supplementary Table 1). The protocol has been registered in Prospero. The registration number is CRD42021278172, and the protocol is available from https://www.crd.york.ac.uk/prospero/display_record.php?ID=CRD42021278172.

## Results

The initial search yielded 376 studies, of which 17 were further assessed in the full text phase. Six RCTs were found, and of these, two were excluded: in the study by Ughasoro et al., randomization was not stratified, and children with ABO immunizations and septic newborns were included [23]; Rezaie et al. included only neonates with G6PD deficiency [24]. Four RCTs were included from the initial search [18, 20–22], and two additional RCTs were found from the references of included articles and included in the systematic review and meta-analysis [17, 19] (Fig. 1).

The six included studies had a total of 880 neonates. Five studies were conducted in Iran and one in Egypt. Four studies used UDCA 10 mg/kg divided into two daily doses, and two studies used 15 mg/kg divided into two daily doses. The inclusion and exclusion criteria in the selected studies were practically identical. Funding sources were not reported in four of the studies, and conflicts of interest were not reported by the authors in two studies (Table 1). Background characteristics of the study populations in the included studies are reported in Table 2. Only one study reported the gestational age of the neonates, and one study did not report any background information.

**Table 1:** Characteristics of included studies

Study	Country	Study period	Blinding	Placebo	Participants, *n*	Dose of UDCA	Inclusion criteria	Exclusion criteria	Funding	Conflict of interest
Hassan et al. [17]	Iran	2014–2015	Unknown	No	200	10 mg/kg per d in two doses	Normal birthweight, age 3–7 d, breast-fed, total bilirubin 14–20 mg/dL, direct bilirubin < 2 mg/dL	Rh or ABO incompatibility, prematurity, sepsis or maternal diabetes	Not reported	Not reported
Honar et al. [18]	Iran	2013	Double-blind	Yes	80	10 mg/kg per d in two doses	Birth weight 2500–4000 g, breast-fed, gestational age 3841 wk, being > 3 d old, total bilirubin level 14–20 mg/dL, direct bilirubin level < 2 mg/dL	Rh or ABO incompatibility, G6PD defiency, conjugated hyperbilirubinemia, speticemia, diseases leading to hyperbilirubinemia (crigler-najjar syndrome, gilbert syndroSme, hypo/hyperthyroidism, liver diseases), prematurity, maternal diabetes	Public funding	None to report
El-Gendy et al. [20]	Egypt	2016–2017	Not blinded	No	100	10 mg/kg per d in two doses	Aged 3 d or more, weighed 2.5–4 kg, total bilirubin 14–20 mg/dL	Prematurity, severe hemolysis, sepsis, or cholestasis	No funding received	None to report
Shahramian et al. [19]	Iran	2017	Double-blind	No	200	15 mg/kg per d in two doses	Birth weight of 2.5 to 4 kg, breast-fed, gestational age 38–41 wk, age 3–5 d, total bilirubin level 12–22 mg/dL, direct bilirubin level < 2 mg/dL	ABO and Rh incompatibility, G6PD deficiency, direct hyperbilirubinemia, septicemia, and diseases leading to hyperbilirubinemia (Crigler-Najjar syndrome, Gilbert syndrome, hypo/hyperthyroidism, liver diseases), prematurity, maternal diabetes	Not reported	None to report
Akefi et al. [22]	Iran	2017–2018	Double-blind	Not specified	220	10 mg/kg per d in two doses	Weight of 2500–4000 g, breast milk fed, gestational age: 37–41 week, age over 48 h, total bilirubin 14–20 and direct bilirubin < 1 mg/dL	ABO and RH incompatibility, septicemia or having diseases resulting in indirect hyperbilirubinemia including crigler–najjar syndrome, Gilbert, hypothyroidism, preterm neonates, neonates with hemolysis or G6PD deficiency, maternal diabetes low hemoglobin and weight loss > 10%	Not reported	None to report
Gharehbaghi et al. [21]	Iran	2017	Double-blind	Yes	80	15 mg/kg per d in two doses	Birth weight > 2500 g, gestational age > 35 wk, total bilirubin level 14–25 mg/dL, direct bilirubin level < 2 mg/dL	Rh or ABO incompatibility (with positive direct coombs test), G6PD deficiency, direct hyperbilirubinemia, sepsis, crigler-najjar syndrome, thyroid disorders, hepatic diseases, maternal diabetes	Not reported	Not reported

*UDCA* ursodeoxycholic acid, *G6PD* glucose-6-phosphate dehydrogenase

**Table 2:** Background characteristics of study populations in included studies

Study	Birth weight (kg), mean (SD)		Gestational age (wk), mean (SD)		Gender (female), %		Age at the time of initiation of phototherapy (d), mean (SD)		Mean bilirubin level at the start of the phototherapy (mg/dL), mean (SD)	
	UDCA	Control	UDCA	Control	UDCA	Control	UDCA	Control	UDCA	Control
Hassan et al. [17]	3.1 (0.4)	3.1 (0.4)	N/A	N/A	44	49	5.4 (1.4)	5.3 (1.5)	16.3 (1.7)	16.5 (2.9)
Honar et al. [18]	2.97 (0.29)	2.99 (0.31)	N/A	N/A	53	55	3.7 (1.0)	3.6 (1.0)	15.9 (1.7)	16.3 (1.5)
El-Gendy et al. [20]	N/A	N/A	N/A	N/A	40	48	4.9 (1.4)	4.9 (1.6)	16.5 (1.4)	16.4 (1.5)
Shahramian et al. [19]	N/A	N/A	N/A	N/A	N/A	N/A	N/A	N/A	15.79 (2.18)	16.89 (2.49)
Akefi et al. [22]	N/A	N/A	N/A	N/A	47	56	5.3 (2.9)	4.9 (2.1)	16.85 (2.4)	15.75 (2.6)
Gharehbaghi et al. [21]	2.96 (0.56)	3.19 (0.43)	38	39	N/A	N/A	5.1 (2.5)	5.9 (2.5)	19.33 (2.51)	19.76 (2.64)

*UDCA* ursodeoxycholic acid, *SD* standard deviation, *N/A* not applicable

### Risk of bias

**Fig. 2:**
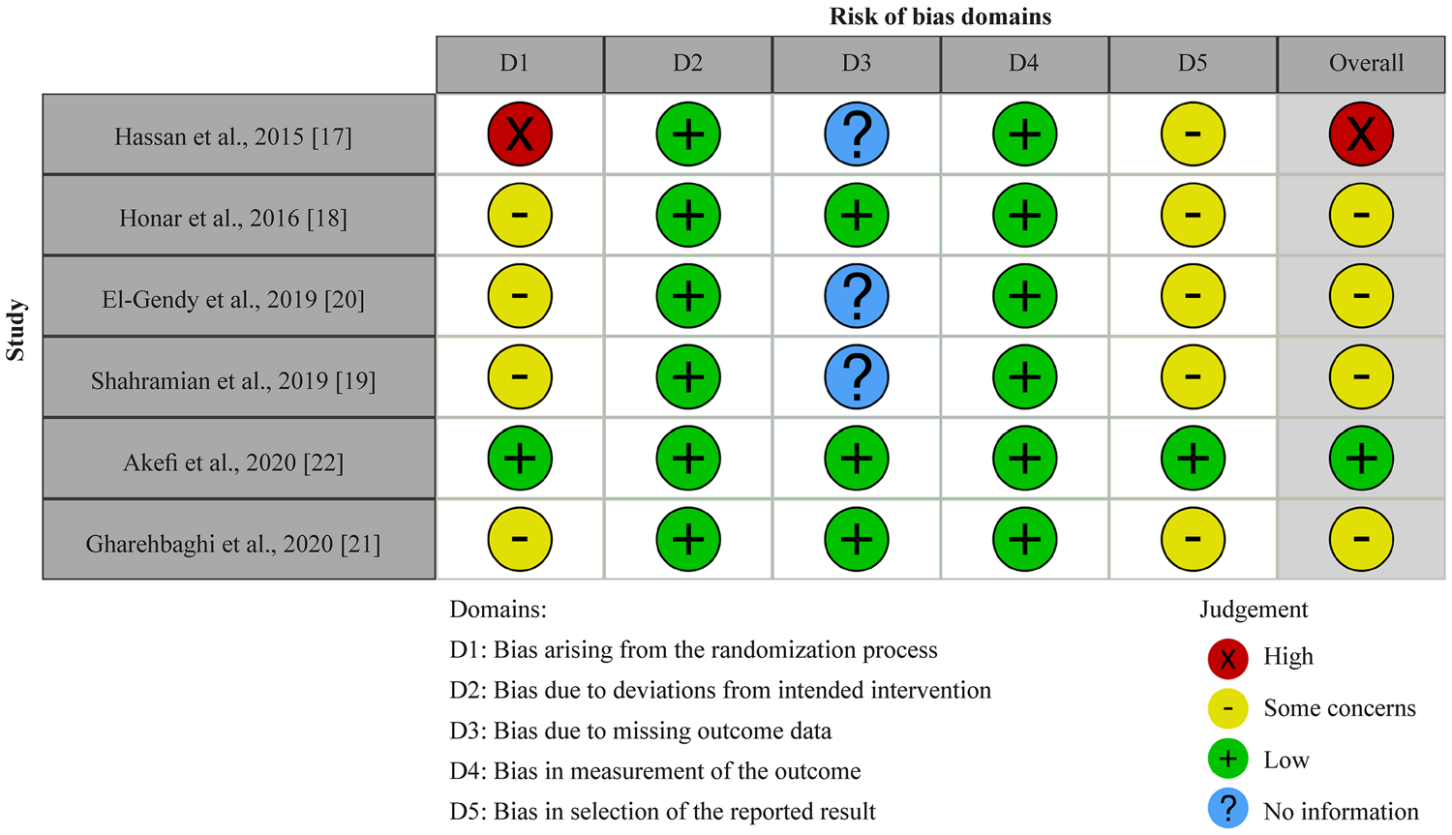
Risk of bias summarized in Cochrane risk of bias two format

The risk of bias was assessed in five domains and overall. Overall, five of the studies had some concerns about the risk of bias assessment. One study had a high risk of bias due to randomization, and four of the studies did not report any adverse events between groups, leading to concern about bias in the selected reported results (Fig. 2).

### Bilirubin level changes during the first 24 hours

**Fig. 3:**
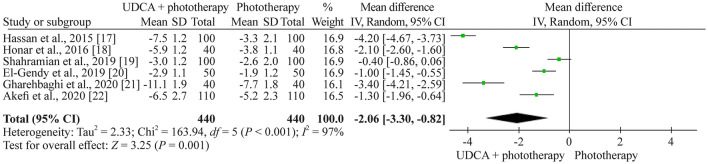
Forest plot of mean bilirubin decrease (mg/dL) during the first 24 hours after the initiation of phototherapy. Random effect model reported as mean difference with 95% CIs. *CI* confidence intervals, *UDCA* ursodeoxycholic acid, *SD* standard deviation

The mean decrease of total bilirubin during the first 24 hours in the included six studies (880 neonates) ranged from 2.5 to 11.1 mg/dL in the UDCA + phototherapy group and from 1.9 to 7.7 mg/dL in the phototherapy group. The weighted mean difference in total bilirubin decrease in the random effect model was 2.06 mg/dL [95% confidence interval (CI) 0.82–3.30], favoring the UDCA + phototherapy (Fig. 3). We ranked the quality of evidence as low (Table 3).

**Table 3:** Grading of recommendations assessment, development and evaluation assessment of evidence

Variables	Included studies	No. of participants	Quality assessment					
			Risk of bias	Inconsistency	Indirectness	Imprecision	Publication bias	Quality of evidence
Total bilirubin at 24 h	6	880	Serious limitations: blinding not performed (second studies), unclear allocation (second studies), randomization not described (first study), no placebo (fourth studies), background characteristics not presented (first study)	No serious limitations	Direct	No serious limitations	Undetected	Low
Mean decrease in bilirubin during the first 24 h	6	880	Serious limitations: blinding not performed (second studies), unclear allocation (second studies), randomization not described (first study), no placebo (fourth studies), background characteristics not presented (first study)	No serious limitations	Direct	No serious limitations	Undetected	Low
Phototherapy duration	5	780	Serious limitations: blinding not performed (second studies), unclear allocation ( second studies), randomization not described (first study), no placebo (fourth studies)	No serious limitations	Direct	No serious limitations	Undetected	Low
Possible side effects	6	880	Serious limitations: only one study reported adverse outcomes and side effects	N/A	Not applicable	Not applicable	Serious limitations	Very low

### Phototherapy duration

**Fig. 4:**
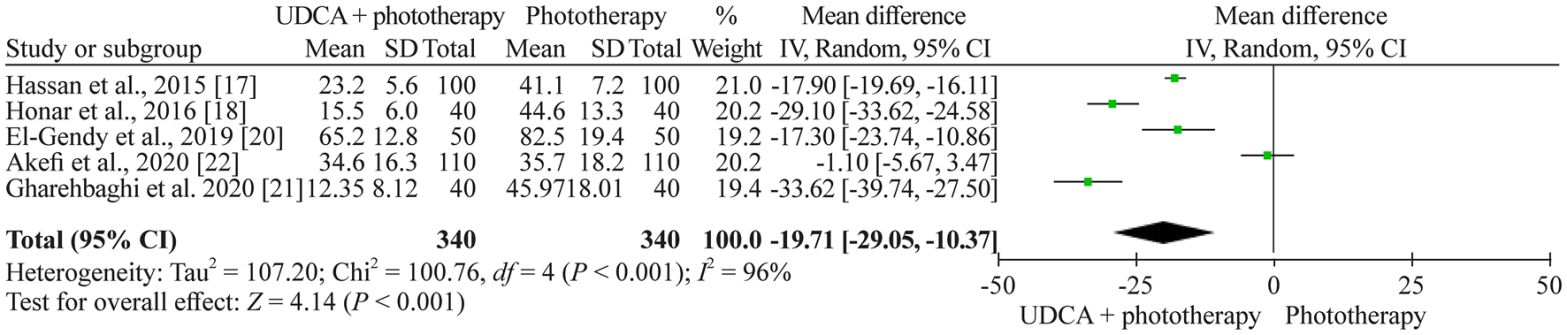
Forest plot of phototherapy treatment duration (in hours). Random effect model reported as mean difference with 95% CIs. *CI* confidence intervals, *UDCA* ursodeoxycholic acid, *SD* standard deviation

Five studies (780 neonates) reported the overall duration of phototherapy. The duration range varied from 12.3 to 65.2 hours in the UDCA + phototherapy group and from 41.1 to 82.5 hours in the phototherapy group. The weighted mean difference in phototherapy duration in the random effect model was 19.7 hours (95% CI 10.4–29.1), favoring the UDCA + phototherapy group (Fig. 4). We ranked the overall quality of evidence as low (Table 3).

### Possible side effects and adverse outcomes

Only one study discussed possible side effects and stated that these were not detected in either group. None of the studies underwent follow-up after discharge. The evidence regarding possible side effects and adverse outcomes was very low (Table 3).

## Discussion

Six RCTs with 880 neonates demonstrated that neonates that received UDCA together with phototherapy was effective in reducing total serum bilirubin levels during the first 24 hours. Five RCTs with 780 neonates showed that UDCA combined with phototherapy was effective and decreased the phototherapy duration by nearly 20 hours compared to standard phototherapy.

The decrease of the total bilirubin level during the first 24 hours of treatment together with the 20-hour reduction in the total duration of phototherapy are potentially clinically significant and beneficial results for patients. These reductions would most likely decrease the rates of acute [10, 11] and long-term harms and adverse effects related to phototherapy [12–16]. The shorter hospital stay could potentially decrease costs related to neonatal hyperbilirubinemia and enable the relocation of healthcare resources. Previous reports have stated that neonates requiring phototherapy have more problems with breastfeeding [35, 36]. It can be speculated that the shortened phototherapy and hospital stays might help to improve breastfeeding rates. This could produce additional value for these neonates [37], but this issue was not evaluated in the original papers. There are no previous meta-analyses on this topic, and therefore, our results cannot be compared to previous reports.

The optimal dose of UDCA remains unsure, as two of the studies used 15 mg/kg daily dose and four studies used 10 mg/kg daily dose. We did not perform any subgroup analysis based on the different doses as it was not preplanned. We observed that the studies with a higher dose [19, 21] showed similar results compared to the studies using smaller doses of UDCA. The optimal dose with the best benefit/harm ratio remains to be determined as majority of the studies included in this meta-analysis did not report any adverse effects.

We had a few deviations from the original protocol. First, we were prepared to use standardized mean differences, as we hypothesized that the studies would not have used the same outcome measure scale. We did not include the use of mean difference in the protocol, but this is a minor deviation. Second, we wanted to analyze adverse outcomes (neonatal mortality and return rates to hospital), but none of the included studies reported these. Third, we wanted to perform a subgroup analysis on preterm neonates, but none of the studies reported these data, and therefore, this was not possible.

The limitations of this review are largely those of the primary studies. As four of the studies were not placebo-controlled [17–21] and one of these did not comment on the blinding at all [17], the results presented here are vulnerable to bias. Furthermore, only one of the included studies reported adverse outcomes [22], and none of the studies reported rehospitalization rates. In addition, the population characteristics were reported incompletely, which limits the generalizability of the results. Two studies did not state the cutoff bilirubin level to stop phototherapy [20, 21]. All the included studies were conducted in relatively small geographical areas (Iran [17–19, 21, 22] and Egypt [20]). Due to genetic factors related to bilirubin metabolism, these results may not be valid in other populations. Furthermore, the included studies had some variation in the exclusion criteria, as two studies did not exclude G6PD patients and overall, the exclusion criteria were not as strict in these two studies [17, 20]. All the studies excluded preterm neonates, which means that these results cannot be generalized to treatment of preterm neonates. UDCA is an off-label drug in newborns and children in Europe and North America. Thus, more studies on its pharmacokinetics and pharmacodynamics, including safety, are needed prior to its implementation into standard treatment of newborns at any indication. None of these studies provided any potential cost-effectiveness analyses. As all the studies excluded neonates with significant hemolysis, we do not know if UDCA would prevent the need for transfusion, for example. The risk of bias was assessed to be moderate in four of the studies and high in one, and only one study had a low risk of bias. The study with the lowest risk of bias stated that UDCA would not bring additional value to standard phototherapy [22]. These concerns should be noted when interpreting the results of our systematic review.

In conclusion, we found low-quality evidence that UDCA is effective as an adjuvant treatment with phototherapy in neonatal hyperbilirubinemia. UDCA decreases the duration of phototherapy by nearly 20 hours, which is a clinically significant finding that would benefit patients and families. Mean bilirubin levels decreased more rapidly during the first 24 hours. Studies in different geographical locations with double-blinding and placebo-controlling are needed with pharmacological, cost-effectiveness and safety analyses before the use of UDCA can be considered a potential option in the standard care of neonatal hyperbilirubinemia.

## Supplementary Information

Below is the link to the electronic supplementary material.

Supplementary file 1 (DOCX 30 kb)

## Data Availability

All data used in this study is available upon request from the corresponding author.

## References

[1] Mitra S, Rennie J (2017). Neonatal jaundice: aetiology, diagnosis and treatment. Br J Hosp Med.

[2] Woodgate P, Jardine LA (2015). Neonatal jaundice: phototherapy. BMJ Clin Evid.

[3] Kuzniewicz MW, Escobar GJ, Newman TB (2009). Impact of universal bilirubin screening on severe hyperbilirubinemia and phototherapy use. Pediatrics.

[4] Newman TB, Wickremasinghe AC, Walsh EM, Grimes BA, McCulloch CE, Kuzniewicz MW (2016). Phototherapy and risk of type 1 diabetes. Pediatrics.

[5] National Institute for Health and Care Excellence. Jaundice in newborn babies under 28 days. 2010. https://www.nice.org.uk/guidance/cg98. Accessed 25 Oct 2021. 32011839

[6] American Academy of Pediatrics Subcommittee on Hyperbilirubinemia (2004). Management of hyperbilirubinemia in the newborn infant 35 or more weeks of gestation. Pediatrics.

[7] Cremer R, Perryman PW, Richards DH (1958). Influence of light on the hyperbilirubinaemia of infants. Lancet.

[8] Itoh S, Okada H, Kuboi T, Kusaka T (2017). Phototherapy for neonatal hyperbilirubinemia. Pediatr Int.

[9] Kumar P, Chawla D, Deorari A (2011). Light-emitting diode phototherapy for unconjugated hyperbilirubinaemia in neonates. Cochrane Database Syst Rev.

[10] Maisels MJ, McDonagh AF (2008). Phototherapy for neonatal jaundice. N Engl J Med.

[11] Stokowski LA (2011). Fundamentals of phototherapy for neonatal jaundice. Adv Neonatal Care.

[12] Wickremasinghe AC, Kuzniewicz MW, Grimes BA, McCulloch CE, Newman TB (2016). Neonatal phototherapy and infantile cancer. Pediatrics.

[13] Auger N, Laverdière C, Ayoub A, Lo E, Luu TM (2019). Neonatal phototherapy and future risk of childhood cancer. Int J Cancer.

[14] Oláh J, Tóth-Molnár E, Kemény L, Csoma Z (2013). Long-term hazards of neonatal blue-light phototherapy. Br J Dermatol.

[15] Maimburg RD, Olsen J, Sun Y (2016). Neonatal hyperbilirubinemia and the risk of febrile seizures and childhood epilepsy. Epilepsy Res.

[16] Newman TB, Wu YW, Kuzniewicz MW, Grimes BA, McCulloch CE (2018). Childhood seizures after phototherapy. Pediatrics.

[17] Hassan AM, Abdulrahman A, Husain RA (2015). Effect of ursodeoxycholic acid in lowering neonatal indirect hyperbilirubinemia: a randomized controlled trial. Merit Res J Med Medical Sci.

[18] Honar N, Ghashghaei Saadi E, Saki F, Pishva N, Shakibazad N, Hosseini Teshnizi S (2016). Effect of ursodeoxycholic acid on indirect hyperbilirubinemia in neonates treated with phototherapy. J Pediatr Gastroenterol Nutr.

[19] Shahramian I, Tabrizian K, Ostadrahimi P, Afshari M, Soleymanifar M, Bazi A (2019). Therapeutic effects of ursodeoxycholic acid in neonatal indirect hyperbilirubinemia: a randomized double-blind clinical trial. Arch Anesth Crit Care.

[20] El-Gendy FM, Bahbaha WA, Al Kaforyb EE (2019). Effect of ursodeoxycholic acid on indirect hyperbilirubinemia in neonates treated with phototherapy. Menoufia Med J.

[21] Gharehbaghi MM, Sani AM, Refeey M (2020). Evaluating the effects of different doses of ursodeoxycholic acid on neonatal jaundice. Turk J Pediatr.

[22] Akefi R, Hashemi SM, Alinejad S, Almasi-Hashiani A (2020). The effect of ursodeoxycholic acid on indirect hyperbilirubinemia in neonates treated with phototherapy: a randomized clinical trial. J Matern Fetal Neonatal Med.

[23] Ughasoro MD, Adimorah GN, Chukwudi NK, Nnakenyi ID, Iloh KK, Udemba CE (2019). Reductive effect of ursodeoxycholic acid on bilirubin levels in neonates on phototherapy. Clin Exp Gastroenterol.

[24] Rezaie M, Gholami R, Jafari M, Haghighinejad H (2021). Evaluating the effect of ursodeoxycholic acid on total bilirubin of neonates with glucose-6-phosphate dehydrogenase deficiency complicated by indirect hyperbilirubinaemia. J Paediatr Child Health.

[25] Ovadia C, Sajous J, Seed PT, Patel K, Williamson NJ, Attilakos G (2021). Ursodeoxycholic acid in intrahepatic cholestasis of pregnancy: a systematic review and individual participant data meta-analysis. Lancet Gastroenterol Hepatol.

[26] van der Schoor LWE, Verkade HJ, Bertolini A, de Wit S, Mennillo E, Rettenmeier E (2021). Potential of therapeutic bile acids in the treatment of neonatal hyperbilirubinemia. Sci Rep.

[27] Willot S, Uhlen S, Michaud L, Briand G, Bonnevalle M, Sfeir R (2008). Effect of ursodeoxycholic acid on liver function in children after successful surgery for biliary atresia. Pediatrics.

[28] Jacquemin E, Hermans D, Myara A, Habes D, Debray D, Hadchouel M (1997). Ursodeoxycholic acid therapy in pediatric patients with progressive familial intrahepatic cholestasis. Hepatology.

[29] Spagnuolo MI, Iorio R, Vegnente A, Guarino A (1996). Ursodeoxycholic acid for treatment of cholestasis in children on long-term total parenteral nutrition: a pilot study. Gastroenterol.

[30] Higgins JP, Altman DG, Gøtzsche PC, Jüni P, Moher D, Oxman AD (2011). The cochrane collaboration’s tool for assessing risk of bias in randomised trials. BMJ.

[31] McGuinness LA, Higgins JPT (2021). Risk-of-bias visualization (robvis): an r package and shiny web app for visualizing risk-of-bias assessments. Res Synth Methods.

[32] Guyatt GH, Oxman AD, Vist GE, Kunz R, Falck-Ytter Y, Alonso-Coello P (2008). Grade: an emerging consensus on rating quality of evidence and strength of recommendations. BMJ.

[33] Higgins JPT, Thomas J, Chandler J, Cumpston M, Li T, Page MJ, Welch VA, editors. Cochrane handbook for systematic reviews of interventions version 6.2 (updated February 2021). Cochrane. 2021. Accessed Oct 2021. https://training.cochrane.org/handbook. Accessed 25 Oct 2021.

[34] Moher D, Liberati A, Tetzlaff J, Altman DG (2009). Preferred reporting items for systematic reviews and meta-analyses: the prisma statement. BMJ.

[35] Chiu YW, Cheng SW, Yang CY, Weng YH (2021). Breastfeeding in relation to neonatal jaundice in the first week after birth: parents' perceptions and clinical measurements. Breastfeed Med.

[36] Kovaric K, Cowperthwaite M, McDaniel CE, Thompson G (2020). Supporting breastfeeding in infants hospitalized for jaundice. Hosp Pediatr.

[37] Binns C, Lee M, Low WY (2016). The long-term public health benefits of breastfeeding. Asia Pac J Public Health.

